# New York Correspondence

**Published:** 1871-09

**Authors:** C. A. Simpson

**Affiliations:** New York


### NEW YORK CORRESPONDENCE.

New York, September 22d, 1871.

*Editors Medical and Surgical Journal,*—I shall give some brief notes of cases treated at the clinics of the hospitals for diseases of the Eye and Ear, in this city, which I hope will not be without interest to some of the readers of the "Journal."

There are, in the city, four such Infirmaries, at which, during the past year, more then fourteen thousand patients, suffering from various diseases of the eyes and ears, have been prescribed for.

Clinics are held every day for the benefit of the poor, at the Infirmaries, with the best men in the city in attendance—such as Roosa, Agnew, Noyes, Althof, etc.

By far the greater proportion of ophthalmic cases in a large and densely populated city as this, occur among the poorer classes, from over-crowding and consequent foul atmosphere, bad and insufficient food, syphilis, scrofula, etc.

These are not only most fruitful sources of ophthalmic diseases, but they prove most inveterate obstacles in the treatment of these diseases at the public dispensaries. These are circumstances, however, over which the surgeon has no control, and they frequently thwart the most patient and systematic treatment, from which he should otherwise expect the happiest results.

The affections most frequently met with among the patients who present themselves for treatment, are granular conjunctivitis and keratitis. They form, probably, more than two-thirds of all the cases treated. The granular lids are treated by applications of sulphate of copper, alum, nitrate of silver, to the granulations every other day. The keratitis, which so frequently accompanies granular lids, is treated by instillation of sulphate of atropine (gr. ij to iv to water ℥ ij.)

There have been two cases—one a boy only three years of age, and the other a boy of twelve—with a small sore on the inner or conjunctival surface of the lower lid, having all the appearance of chancres. The parotids and some glands of the neck were enlarged. There was no sore on the penis, nor was there any history connected with the cases. It may be left to conjecture, if these were chancres, how they were produced in that abnormal situation.

There have been several cases of dyptheritic conjunctivitis, which are interesting from the fact that the disease is seldom seen in this country—though frequently met with in Europe—and the peculiar obstinacy with which it usually runs its course, regardless of all manner of treatment; besides, it is one of the most contagious of ophthalmic diseases.

CASES TREATED AT THE MANHATTAN EYE AND EAR HOSPITAL.

Mrs. B.—æt. 35—(neuro-retinitis nephretica)—states that seven years ago she was sick in bed for six months. (She had acute Bright's disease.) Says she never noticed that her sight was failing until about ten weeks ago.

Cornea and lids clear. R eye V 1-10; L eye V 1-5. Ophthalmoscope shows large white patches, interspersed with strings of pigment, in the region of both maculæ; choroidal exudations in other portions of fundus.

Treatment.—Hydrarg bichlor, gr. 1-32.
Sig: Three times a day.

John R.—æt. 48—states that three and a half years ago he had a wound in left eye from a pick, and that he has been losing his sight gradually since then. The pupil of R eye is closed from old inflammation. There is general opacity of L eye, in which a previous iridectomia had been done. Græfi's knife was entered near edge of cornea of R eye, the point passed through the iris, and a small piece excised. The knife was then withdrawn, and the forceps passed in through the corneal wound, the detached piece of iris siezed and extracted. There was some blood and a bubble of air left in anterior chamber. Patient had had a cough for several days before the operation, and for several days thereafter was very sick, with a severe attack of pleuro-pneumonia; notwithstanding which, however, the case went on well, and in fifteen days from the date of admission he was discharged, being able to count fingers at ten feet, and still improving.

Joseph H.—æt. 60—(cataract in both eyes)—states that eighteen months ago he was exposed to the bright reflection from the snow, since then light has been disagreeable to his eyes; lost the power of reading six months ago; could see to go about until four weeks ago; has never had pain or redness, nor luminous spectres in the eyes. Cataract in both eyes well advanced; pupils active; in dark room sees light of candle in all parts of visual field with both eyes. Radial artery at wrist felt to be atheromatous. He was put under the influence of a mixture of chloroform i, to ether iij, (ether alone is almost always used,) and Dr. Agnew performed the operation of extraction on R eye, by Gæfi's periph linear, upwards. There was no vitreous lost, nor was there any other accident during the operation. Slight vomiting, with very little straining, after the operation. Strips of isinglass-plaster were put over each eye, and covered with Dr. Agnew's black silk dressing. Next day felt as if dirt was in the eye, otherwise no bad symptoms. Eye carefully cleansed, without raising the lids, and a drop of solution of atropine instilled into eye. On fifth day bandage removed; very little secretion; corneal wound healed. Discharged a few days after, V 20-70, and reads Snellen 3½, at one foot.

James C.—æt. 21—blown up with gunpowder; one eye entirely destroyed; sympathetic ophthalmia feared in sound eye, and an operation determined upon. He was put under the influence of ether, and the lids held wide apart with the spring speculum. The globe was then steadied with the forceps, while a circular division of the conjunctiva was made close to the edge of the cornea. The external rectus was then seized with the forceps, and its tendon snipped through; an assistant seizes the cut tendon and draws the eye inwards; with the trabismus hook, the superior rectus, oblique muscles and inferior rectus were raised from the ball, the curved scissors passed beneath, and their tendons cut through; the ball was then thrust forward, the optic nerve cut, and then the inferior rectus. There was very slight bleeding.

Treatment.—Cold water applications.

Remarks.—This is the operation of enucleation, devised by Bonnet and O'Farrel, in 1841. The advantages claimed for this operation over the older method, are, that the eye is removed from the ocular capsule without any injury to, or interference with the cellular tissue of the orbit, or a division of the outer commissure of the eye-lids; that the muscles are divided quite close to their insertion into the sclerotic; that nearly the whole of the conjunctiva is preserved; that only a few vessels are divided; and that a better stump is left for an artificial eye.

The following are the causes for which Pagenstecher has found enucleation necessary:

1st. Traumatic irido-choroiditis, occasioned by—

(*a*) Lesion of iris, resulting from its being nipped between the edge of the wound.

(*b*) Lesion of the choroid.

(*c*) Suppurative choroiditis, or suppuration in the vitrious.

(*d*) Presence of foreign body in the eye.

(*e*) Lesion of the capsule of the lens.

(*f*) Choroiditis after reclination, or depression of the lens.

2d. Incipient exudative choroiditis and hæmorrhage from the choroid.

3d. Processes leading to staphyloma.

4th. Extensive separation of the retina.

5th. Tumors developing from choroid or sclerotic.

6th. Formation of bone within the choroid.

C. A. Simpson, S. R. C. S. E.

